# Severe osteoporosis as atypical presentation of hereditary hemochromatosis

**DOI:** 10.1002/ccr3.6396

**Published:** 2022-10-08

**Authors:** Georgiana Cristina Taujan, Laura Iconaru, Mihaela Rosu, Olga Ana Kosmopoulou, Ioana Blerta Papadopoulou, Felicia Baleanu

**Affiliations:** ^1^ Department of Endocrinology Centre Hospitalier Universitaire Brugmann Brussels Belgium

**Keywords:** hemochromatosis, iron overload, male osteoporosis, vertebral fractures

## Abstract

Besides important metabolic repercussions, iron overload is reported to be associated with deleterious effects on articulations and bones. We present the case of a male patient diagnosed with severe osteoporosis and vertebral fracture, in whom the evaluation for secondary osteoporosis revealed hereditary hemochromatosis.

## Graphical abstract

Exacerbation of chronic back pain should raise suspicion of vertebral fractures even in men, although severe osteoporosis with vertebral fractures is more common in women. A simple blood test panel can reveal the main cause of osteoporosis.

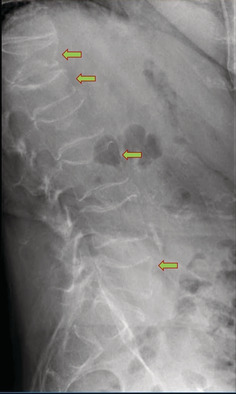

## 1 INTRODUCTION

Hereditary hemochromatosis (HH) is an autosomal recessive disorder with low penetrance and seems to be the most common genetic condition in people of European descent.^1^ It is now more often diagnosed due to genetic testing in context of familial history, high ferritin levels or non‐specific symptoms as fatigue, lethargy, articular, and bone complaints. Organs classically known to be affected by hemochromatosis include the liver, pancreas, heart, thyroid, joints, skin, gonads, and pituitary.

There are few studies reporting a higher prevalence of osteoporosis and vertebral fractures in these patients, especially in men. Although the pathophysiology of this complication is complex and still in process of being revealed,^2^ the targeted treatment remains the same as for the primary osteoporosis, with a significant impact on fracture risk reduction and improvement of quality of life.

## 2 CASE REPORT

**FIGURE 1:**
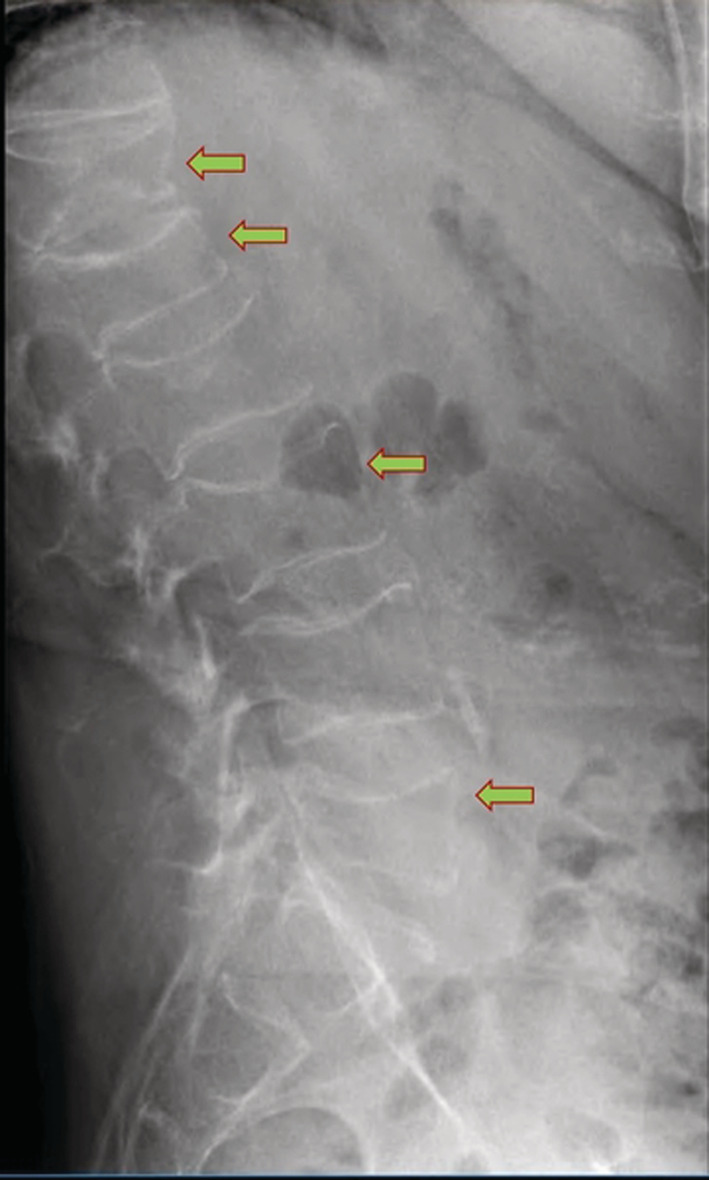
X‐ray with showing multiple vertebral fractures

**FIGURE 2:**
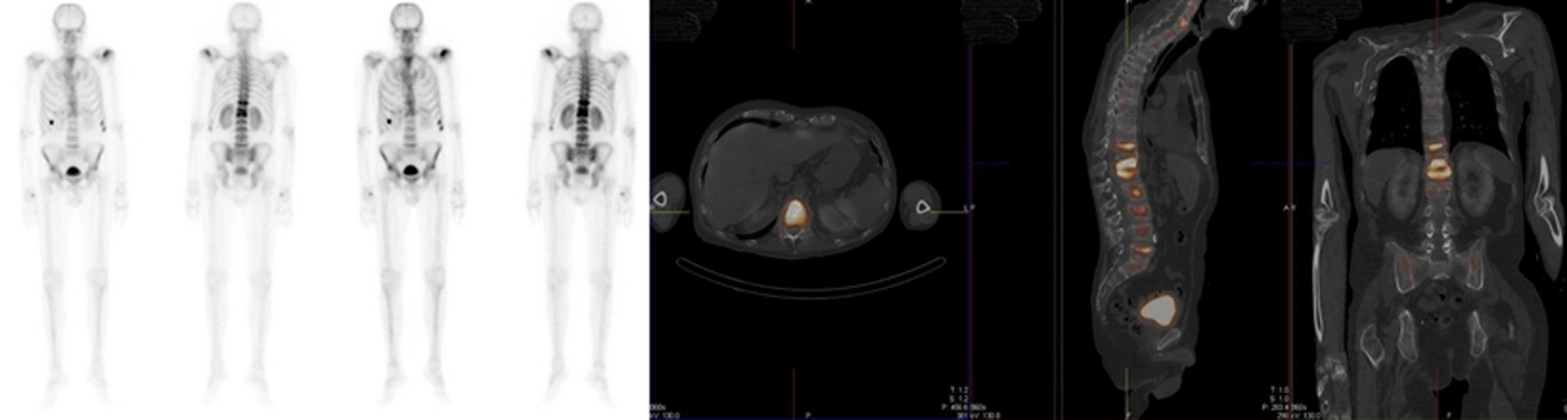
Whole body scintigraphy showing recent vertebral compression fractures at T11, T12, and L1 and semi‐recent factures at the L2, L3, L4, and L5 levels. The scintigraphy also showed rib fractures at the level of the 6th right anterior costal arch and the 7th and 8th left anterior costal arches.

**FIGURE 3:**
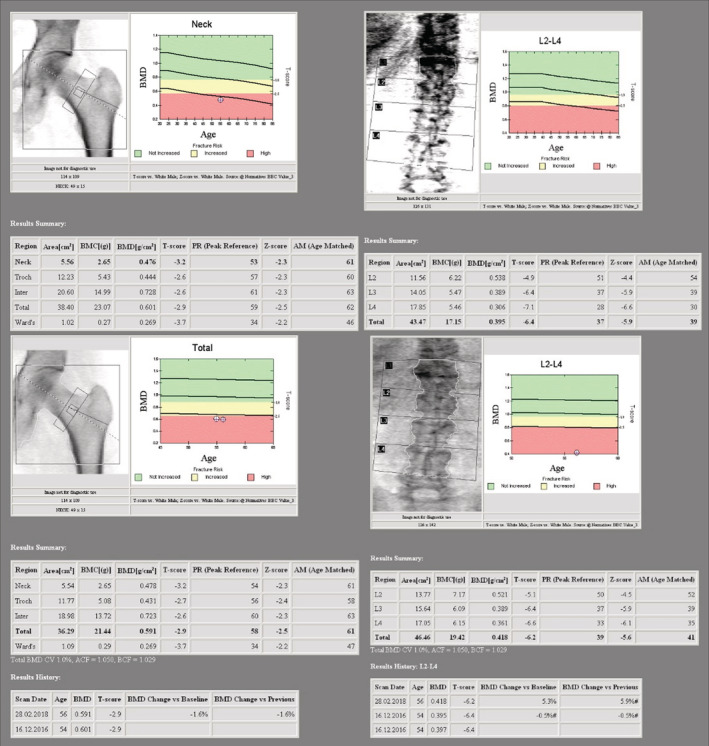
Osteodensitometry before and after 1 year of treatment by phlebotomy, bisphosphonate, calcium, and vitamin D supplementation.

**FIGURE 4:**
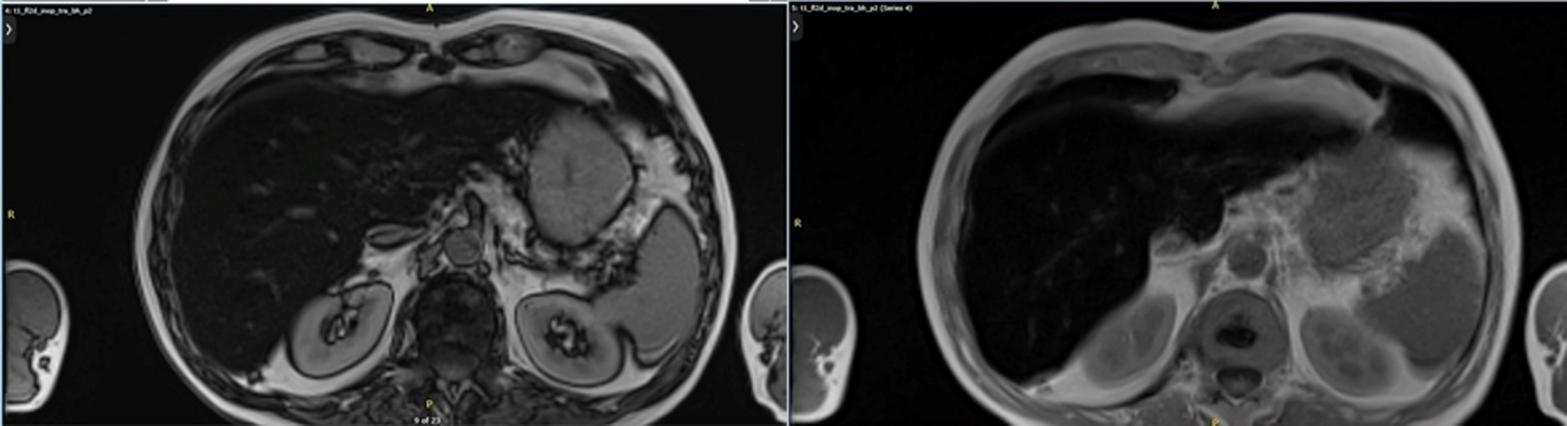
Abdominal MRI showing important hepatic hypointensity and estimating important iron overload at 360 mcmol/gr (VN <36 mcmol/gr).

A 55‐year‐old male patient was referred to our Metabolic Bone Diseases Clinic for severe osteoporosis with multiple vertebral fractures. The patient's medical history was positive for chronic lumbar pain and arterial hypertension, for which he was treated with indapamide, enalapril and lercanidipine. The osteoporosis was incidentally discovered when he presented to the emergency department with severe, invalidating back pain, that started the day after some light gardening, with no history of trauma. The physical examination was unremarkable except for a height loss of 7 cm in the last 5 years. The thoraco‐lumbar X‐ray and lumbar magnetic resonance imaging (MRI) showed a 5‐level severe compression vertebral fractures with collapse of the superior endplates of T11, T12, L2, L3 and L4 (Figure 1). Whole body scintigraphy showed recent T11, T12, and L1 fractures, older L2‐L4 fractures and fractures of the 6th right rib, 7th and 8th left ribs (Figure 2). The initial laboratory evaluation showed high iron and ferritin levels (247 mcg/dl, VN 50–160; 4604 mcg/L, VN 20–300), with high level of transferrin saturation (75%, VN 20–50), mild hepatic cytolysis, glucose intolerance (fasting blood sugar level of 116 mg/dl, HbA1c 4.7%), and vitamin D deficiency (16 ng/ml), with normal calcium and phosphorus and a high‐normal cross‐laps level (459 pg/ml, VN 16–584); protein electrophoresis, IGF1, thyroid, adrenal and gonadal hormones, rheumatological markers, anti‐gliadin, and anti‐transglutaminase antibodies were also normal. The initial osteodensitometry showed a lumbar bone mineral density (BMD) of 0.395 g/cm^2^ with a T‐score of −6.4 standard deviation (SD); at the left femoral neck (FN), the BMD was 0.476 g/cm^2^, with a T‐score of −3.2 and the total left hip BMD was 0.601 g/cm^2^, with a T‐score of −2.9 SD (Figure 3). The diagnosis of severe osteoporosis with multiple vertebral fractures was established. The molecular analysis of the *HFE* gene was performed which came back positive for C282Y pathogenic variant in a homozygote state, confirming therefore the HH diagnosis. The H63D pathogenic variant was negative. Liver MRI showed low signal intensity of liver, estimated using T1 sequences, corresponding to major iron overload at 360 μmol/g (normal values below 36 mcmol/g) (Figure 4). The heart MRI showed no significant iron load at this level.

Iron depletion therapy by phlebotomy was initiated, without the need of any iron chelation therapy. The patient had monthly phlebotomies for almost 18 months, followed by one every 2–3 months. He also received antiresorptive therapy (zoledronate, 5 mg iv per year), calcium and vitamin D supplementation. The osteodensitometry performed after 1 year of treatment, showed a 6% increase in BMD at the lumbar spine compared to the initial evaluation (lumbar BMD of 0.418 g/cm^2^ and T‐score of −6.2 SD; at the FN, the BMD was 0.478 g/cm^2^, with T‐score of −3.2 and total hip BMD was 0.591 g/cm^2^, with T‐score of −2.9 SD) (Figure 3). New vertebral fractures were excluded by vertebral fracture assessment (VFA) and thoraco‐lumbar MRI.

The family history of the patient was positive for osteoporosis in both his mother and his sister. Since they are living abroad, we have no data regarding their iron metabolism and no genetic testing was performed. The patient has one daughter, presenting no symptoms and no pathological laboratory modifications; the genetic screening is scheduled in the near future.

## 3 DISCUSSIONS

HH is a metabolic disease caused by pathogenic variants of different genes and is characterized by increased iron absorption and accumulation in different organs. As the most frequently described genetic variants are caused by mutations in the HFE gene, located on chromosome 6p, HH can be categorized as HFE‐related and non‐HFE related. The first category is by far the most common cause of HH in Western and Nord European populations. However, in Southern Europe, the prevalence of HFE‐unrelated HH is about 30%.^3^, ^4^

More than 90% of subjects with HH are homozygous for the missense mutation of the HFE‐gene that results in the substitution of tyrosine for cysteine at amino acid 282 (C282Y).^5^ This genetic variant is almost absent in the non‐Caucasian populations. Other common variants of HFE‐HH are characterized by the substitution of aspartate for histidine at amino acid 63 (H63D) or cysteine for serine at amino acid 65 (S65C), but these rarely cause iron overload in the absence of C282Y. Also, the compound heterozygous for both the C282Y and the H63D mutations (1%–5% of subjects with HH) will develop a much milder form than the C282Y homozygotes.

The prevalence of C282Y homozygosity is 0.6% in individuals of European descent (in the UK Biobank study) and 0.4% among ethnic Danes; the heterozygosity prevalence was found to be 14.3% in the UK Biobank Study and 14.9% in the Danish population.^5^

Hemochromatosis is known to associate a high risk for osteoporosis, especially in the C282Y homozygous patients, in whom there have been described low‐bone mass, altered microarchitecture and biomechanics and increased incidence of fractures. The mechanisms underlying these changes are complex including increased bone resorption by facilitating osteoclastogenesis and by increasing osteoclast activity, with elevated RANKL/OPG ratio,^6^ decreased osteoblastic differentiation of multipotent mesenchymal cells, and direct inhibition of extracellular matrix mineralization by direct inhibition of hydroxyapatite crystal growth by the iron molecules.^3^, ^6^ Increased oxidative stress also seems to play an important role because metabolically active iron catalyzes the formation of free radicals, which can lead to cell damage and eventually death and also to activation of NF‐kB, a key factor involved in osteoclastogenesis. There have been also described immune alterations consisting in high levels of proinflammatory cytokines TNF‐α and IL‐6, which seem to increase osteoclast formation, promoting thus bone resorbtion.^7^

There are several clinical case reports describing severe osteoporosis in hemochromatosis patients, like the ones published by Eyres et al., Duquenne et al. or Montano et al.^8^, ^9^, ^10^

The first cross‐sectional study that evaluated the prevalence and risk factors of osteoporosis in patient with HH was the one conducted by Sinigaglia et al, in 1997.^11^ They concluded that osteoporosis was observed in 28% of patients and was highly associated with the degree of iron overload.

The second large study was the one conducted by Guggenbuhl et al and was published in 2005.^12^ In this retrospective study, which included 38 men with HH, osteoporosis was found in 34.2% of patients and osteopenia in 78.9%. The main conclusion was that there is significant bone loss in HFE‐related hemochromatosis that cannot solely be explained by hypogonadism or cirrhosis. They have equally observed that BMD at the FN was inversely related to iron hepatic concentration.^13^

Valenti and al., in their retrospective study published in 2009, which included 87 patients with HH, showed that osteoporosis was present in 25.3% of patients and osteopenia in 41.4%, independent of genetic background. 69% of patients without hypogonadism/menopause or cirrhosis, with only iron overload as risk factor, had either osteoporosis or osteopenia.

In HH patients, periodic phlebotomies are the main therapeutic strategy for the underlying disease and chelating iron therapies are very rarely needed. As for the treatment of osteoporosis in a patient with HH is recommended to follow the same guidelines as in patients without hepatic disease, taking into account the individual characteristics. Bisphosphonates, along with calcium and vitamin D supplementation, are most often used. Smoking cessation, ensuring adequate nutrition, high‐impact progressive resistance exercise, maintenance of a healthy body weight, and falls risk reduction are also important aspects of therapeutic strategy.^14^ Besides the specific osteoporosis treatment, periodic phlebotomies have been shown to improve the ferric profile and thus improve osteoblastic function, as shown in by Valenti et al.^4^ in their study, where they described an amelioration of lumbar spine BMD in 66% of a subset comprising the most severely affected patients (32 patients, with a follow‐up 7.4 ± 3 years). However, they pointed out that it is difficult to differentiate whether the beneficial effect of iron depletion was due to iron mobilizations from the bones or to the improvement of hypogonadism.

## 4 CONCLUSIONS

Despite these complex mechanisms, the treatment of hemochromatosis‐associated osteoporosis is similar to other forms of osteoporosis and rapid onset of treatment has a significant impact on lowering the risk of comorbidities by reducing the risk of recurrent fractures.

Although osteoporosis and vertebral fractures are much more common in women, they should be considered as differential diagnosis of acute lumbar pain also in men. Once the diagnosis of osteoporosis is established, evaluation for secondary osteoporosis should be performed and hemochromatosis should be considered. On the other hand, bone mass should be assessed when confronted with a HH patient.

## AUTHOR CONTRIBUTIONS

GT, LI, and FB designed the case report. GT wrote de first draft of the manuscript. MR, OAK, and IBP revised the subsequent versions of the manuscript. All authors read and approved the final version of the paper. IL and FB gave final approval of the version to be published. GT accepts responsibility for the integrity of the data analyses.

## FUNDING INFORMATION

The authors received no financial support for research, authorship, or publication of this article.

## CONFLICT OF INTEREST

All authors state that they have no conflicts of interest.

## ETHICAL APPROVAL

The consent has been obtained from patient after full explanation of the purpose and nature of all procedures used.

## CONSENT

Written informed consent was obtained from the patient to publish this report in accordance with the journal's patient consent policy.

## Data Availability

Data available on request from the authors
